# Strategic donor investments for strengthening condom markets: The case of Zimbabwe

**DOI:** 10.1371/journal.pone.0221581

**Published:** 2019-09-06

**Authors:** Noah Taruberekera, Kumbirai Chatora, Staci Leuschner, Malvern Munjoma, Hardwin Sithole, Sumathi Balasubramanian, Faith Jiyeong Park, Ryan Rego, Andrea Rowan, Kim Longfield

**Affiliations:** 1 Population Services International, Harare, Zimbabwe; 2 Population Services International/Zimbabwe, Harare, Zimbabwe; 3 Population Services International, Washington, DC, United States of America; 4 Databoom, London, United Kingdom; 5 Databoom, Washington, DC, United States of America; University of Maryland College Park, UNITED STATES

## Abstract

**Background:**

Zimbabwe faces an uncertain future for condom funding and potential condom insecurity as international donors prioritize creating more self-sustaining markets and the government identifies how to best ensure access and uptake. We tested the impact of an intensive intervention on demand and supply after a price increase to the social marketed condom, Protector Plus. The study occurred during a deteriorating economy and pressure to reach sustainability quickly. We highlight where strategic donor investments can impact condom programming and markets.

**Methods:**

We randomized ten purposively selected districts in Zimbabwe and assigned them to two study groups to test the impact of an intensive social marketing intervention. To the best of our knowledge, this is the first experimental study conducted within a larger market strengthening context. We tracked sales of Protector Plus and distribution of the public sector condom monthly. We conducted baseline and follow-up surveys among consumers and traders, and used the difference-in-difference method to test the intervention’s impact on condom preferences and brand equity.

**Results:**

Protector Plus sales rebounded to previous levels after the price increase. We detected no significant difference in sales between the experimental and control districts. Among traders, there were no significant differences in brand preference for Protector Plus attributed to the intervention. Among consumers, there was a significant increase in emotional attachment and beliefs about condom efficacy in the experimental districts.

**Discussion:**

Study findings demonstrate where international donor and government investments can impact condom programming and condom markets. Broader findings from the intervention highlight where investments can improve condom coverage, cost recovery, and collaboration between the public, social marketing, and commercial sectors. Strategic investments for strengthening condom markets include: consumer research to segment markets, willingness to pay studies to set price points, distribution system improvements to increase efficiency, intensive demand generation to increase demand and use, market facilitation across sectors, and market intelligence to inform decision making. When a disciplined social marketing approach is used, the market benefits: subsidies can be better targeted, branded products can appeal to the right audiences, and room can be made for the commercial sector to enter the market.

## Introduction

In Zimbabwe, HIV prevalence is estimated to be 13.5% among men and women of reproductive age [1]. Male condoms are an important part of the national HIV prevention strategy and Zimbabwe has historically had one of the strongest condom programs in the southern Africa region. It is one of only five countries to meet or exceed UNFPA’s regional benchmark of 30 male condoms/man/year [2,3,4].

Overall rates of condom use increased significantly during the past two decades for some groups in Zimbabwe. Use increased among non-marital, non-cohabitating partners: from 43% in 1999 to 67% in 2015 for women, and from 70% to 85% for men over the same time period [5,6]. Use in some groups, however, like men with multiple sexual partners, declined [6]. Rates of condom use have also been too low to adequately protect the sexually active population from HIV infection. Additionally, there have been alarming trends in other risk behaviors, like the proportion of men reporting multiple sexual partners, which increased from 11% to 14% between 2010 and 2015 [6]. Trends like this highlight the need for consistent condom promotion and marketing to ensure that all high-risk sexual encounters are protected.

Over the years, international donors have made significant investments in condom programming and Zimbabwe’s condom market. The government of Zimbabwe has supported the distribution of free condoms via the public sector for over 25 years. The goal of the government program has been to prevent HIV among vulnerable populations including young people and key populations by ensuring that these populations are motivated to use condoms, have access to quality condoms, and can use them correctly and consistently.

In the non-profit sector, Population Services International (PSI) has operated the largest condom social marketing (CSM) program in Zimbabwe since 1996 with support from the United States Agency for International Development (USAID) and the United Kingdom’s Department for International Development. The goal of CSM has been to put lifesaving products into the hands of the people who need them by using marketing concepts like consumer insight, product design, appropriate pricing, sales and distribution, and targeted communications [7]. The commercial sector has shown little interest in Zimbabwe’s condom market, likely due to the dominance of public sector distribution and social marketing, and limited profitability: its market share has historically been less than one percent [8].

Today, Zimbabwe faces an uncertain future in terms of condom funding levels, as well as potential condom insecurity. Donors are focusing on the need to create a more self-sustaining condom market. For international donors, a more sustainable condom market means decreasing dependency on donor subsidies and having social marketed condoms operate at cost recovery, while still maintaining sales volumes. For the Zimbabwe government, a more sustainable market would require better targeting of free condoms to groups most in need. A sustainable market would also include more commercial sector players and a broader range of commercial condoms for consumers who can afford them. This means that market actors, like PSI and the government, must employ a total market approach and navigate the tension between cost recovery while still increasing demand for condoms [9,10]. They must also ensure that the appropriate “safety nets” are in place for the poorest populations. Finally, market actors must understand the potential for and contribution of the public, social marketed, and commercial sectors in closing the gap in condom use and decreasing the spread of HIV.

## The condom market in Zimbabwe

**Fig 1:**
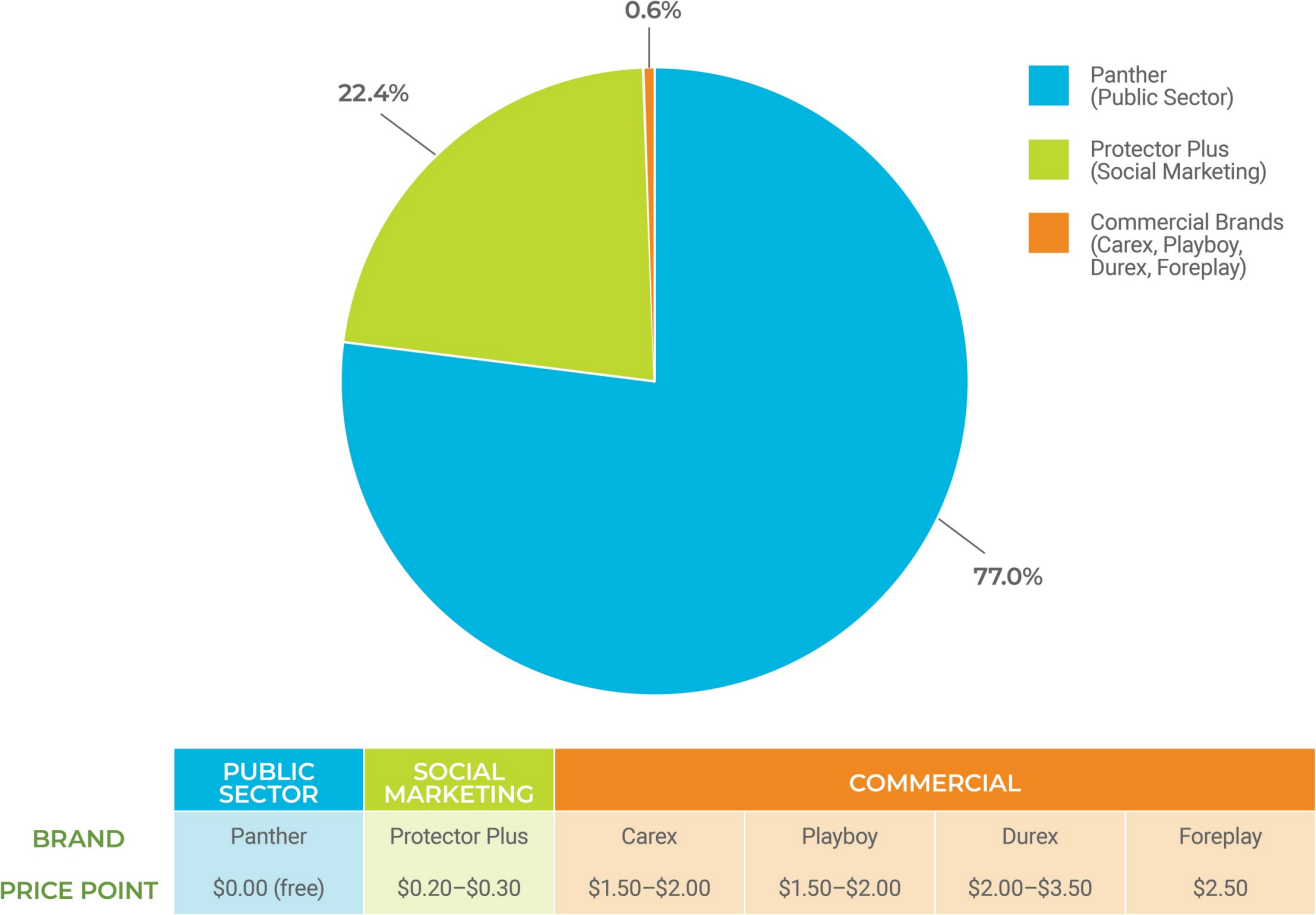
Market share for public, social marketed, and commercial condoms; prices to consumers’ pre-intervention.

In 2016, a total of 113 million condoms were distributed in Zimbabwe [8]. Market share and price points for public, social marketed, and commercial condoms are presented in Fig 1. The public-sector brand, Panther, made up approximately 77% of the market. The social marketed brand, Protector Plus (PP), had 22% market share. The commercial sector was negligible at 1% [8].

Panther condoms are free. In 2016, PP condoms were sold at a price of USD $0.20 for a pack of four PP Original condoms and USD $0.30 for the PP Scented variety. Commercial sector condom prices ranged from USD $1.50/pack of 3 condoms for the Carex brand up to USD $3.50/pack for some varieties of Durex condoms.

## Intervention and methods

Evidence is needed to inform how condom markets can be strengthened, both in terms of financial sustainability and overall demand for condoms. This study tested the effects of an intensive CSM intervention for PP that coincided with a nationwide price increase for PP. We hypothesized that an intensive CSM intervention could generate increased demand for PP that would offset the price increase. To the best of our knowledge, this is the first experimental study conducted within a larger market strengthening context. The aim of this study was to highlight areas where strategic donor investments in market strengthening activities could increase demand for condoms and improve the financial sustainability of condom markets.

## Theory of change

**Fig 2:**
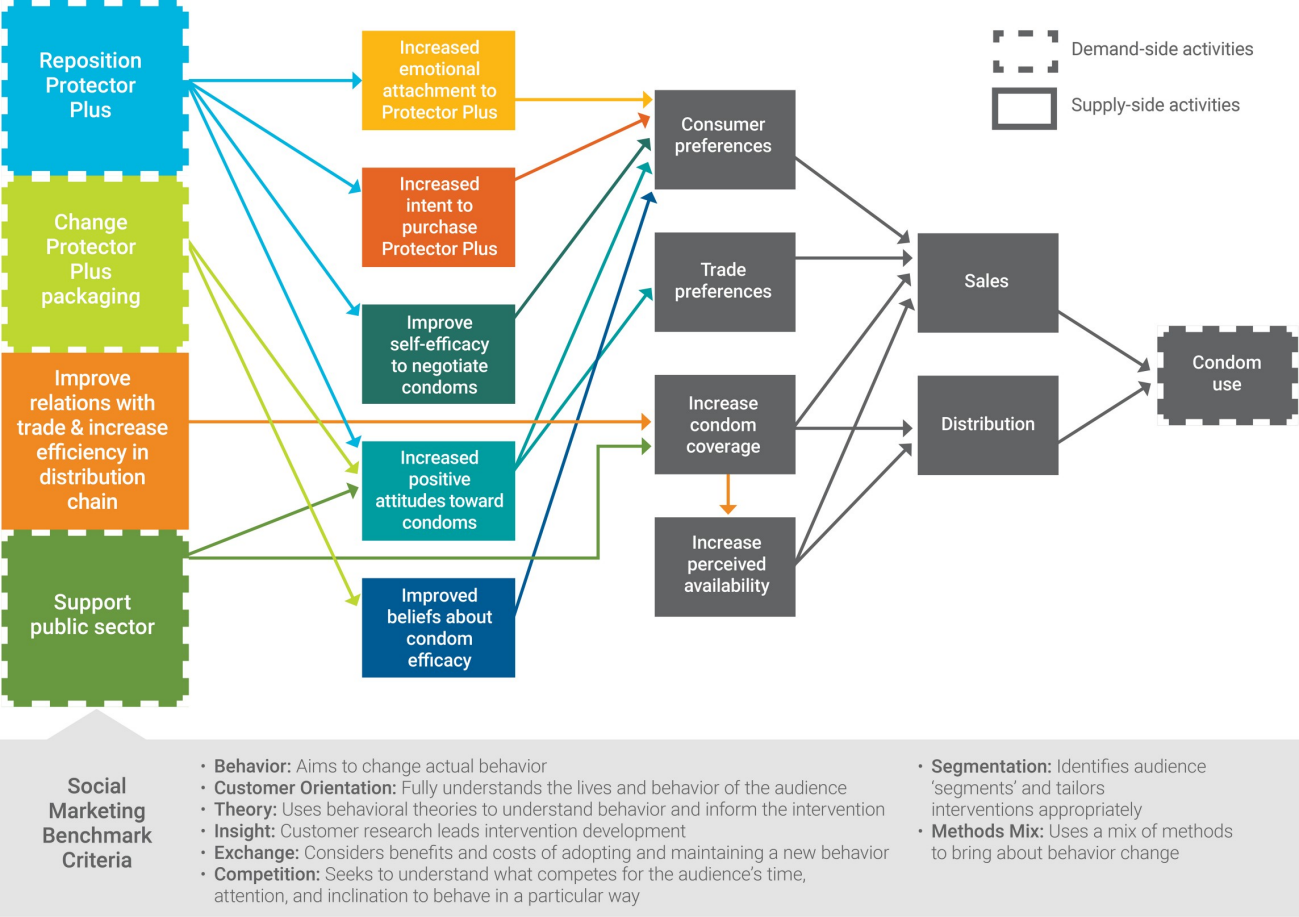
Theory of change.

Drawing on established principles of social marketing, we developed a theory of change (Fig 2) to test if demand generation activities could offset a price increase for PP. The theory also postulated that the right set of interventions could ultimately increase the size of the Zimbabwe condom market and its financial sustainability.

For PP, we hypothesised that (1) even with a price increase, PSI could increase consumers’ emotional attachment to the PP brand, increase their intent to purchase PP, and improve their attitudes and beliefs about condoms by following the social marketing benchmark criteria, repositioning PP, and changing PP packaging. We also hypothesized that (2) by improving trade relations and increasing efficiency in the PP supply chain, PSI could improve the trade’s preferences for PP, PP coverage, and perceptions of PP coverage, despite a price increase. Interventions focused on increasing demand among consumers and the trade and increasing sales for PP, both proxies for future condom use. By having a more intensive CSM intervention in the experimental districts, we believed that increased consumer and trader attachment to PP would lead to a larger increase in condom sales in the experimental, as compared to the control districts.

In the initial study concept, we planned to work closely with the public sector to improve demand for and distribution of Panther condoms. We hypothesised that (3) if PSI supported the Ministry of Health (MOH) to appropriately position Panther and increase overall demand for condoms, consumers would have more positive attitudes toward condoms, higher perceived availability, and the public sector could improve condom coverage for Panther. Repositioning and rebranding Panther, however, involved significantly more time than repositioning PP and executing the study. Therefore, this portion of the experiment was not completed. In the Discussion section of this paper, we share lessons learned about repositioning public sector condoms and considerations for future work in this area.

## Intervention

The intervention consisted of a nationwide price increase, repositioning for PP for both consumers and traders, improvements to distribution and promotion, and improved relationships with the trade. Since PP has been an essential brand in the Zimbabwean condom market since 1996, many of the rebranding and repositioning efforts had to take place nationwide. PSI needed to act responsibly and ensure that Zimbabweans continued to have access to PP. The intervention included the following elements:

- A nationwide price increase for PP: consumers and traders in experimental and control areas all experienced the increase.
- An adequate supply of condoms nationwide: both experimental and control districts had access to an adequate supply of condoms.
- Nationwide improvements to supply-side efficiency and cost recovery: experimental and control districts experienced more focused sales efforts, increased use of stockists and wholesalers, and reduced sales visits from PP agents.
- A repositioning of PP and improvements to its packaging: experimental and control districts received the new version of PP.
- Mass media promotion of PP (via radio and social media) and media bursts to promote PP during key periods of the year, like Father’s Day and Heroes’ Day: experimental and control districts received all mass media promotion.
- Intensive promotional activities to increase product visibility in stores, extra PP promotions, community-level interventions in high-risk venues like bars, and improved relationships with the trade. These intensive activities were conducted only in experimental districts.

Prior to the inception of this intervention, PSI supported a separate willingness to pay (WTP) study to inform the appropriate PP price point for consumers [11]. That study indicated that the optimal price would be $0.50: consumers perceived the USD $0.50 price point as neither too expensive nor too cheap for the social marketed brand. A second WTP study, conducted prior to this study by an external research team, also indicated that PP could tolerate a price increase to USD $0.40 [12]. Based on the results of these WTP studies and the amount of revenue needed for PSI to reach cost recovery for in-country direct operating costs, PSI decided to increase the price of PP to consumers from USD $0.20 to USD $0.50 for a four-pack of PP Original and USD $0.30 to USD $0.50 for a four-pack of PP Scented. The interventions described in this article were conceptualized to enhance willingness to pay among consumers after the PP price increase. This study did not test this formative research, but we offer lessons learned for using WTP research when working in condom markets in the Discussion section.

For traders, raising and standardizing the price for PP would ensure industry average profit margins to eliminate differential pricing for consumers and guarantee set profit margins for wholesalers and retailers. The nationwide PP price increase for traders was from USD $2.50 to USD $7.50 for a dispenser of PP Original, and from USD $5.00 to USD $7.50 for a dispenser of PP Scented. Dispensers of PP Original and PP Scented contain 30 four packs [13].

There were several nationwide improvements to improve supply-side efficiency and increase cost recovery, which were captured in organizational and accounting records. PSI narrowed PP distribution to 35 of 59 high volume districts, which focused sales representatives’ efforts on areas with a higher concentration of commercial distribution networks and higher HIV prevalence. PSI increased the use of stockists and wholesalers (including a designated pharmaceutical retailer) from 160 to 249 as suppliers for small outlets. This reduced the number of sales visits required by PP representatives. Trade discounts for key account customers were also eliminated and PSI reduced the number of PP sales representatives it employed.

**Fig 3:**
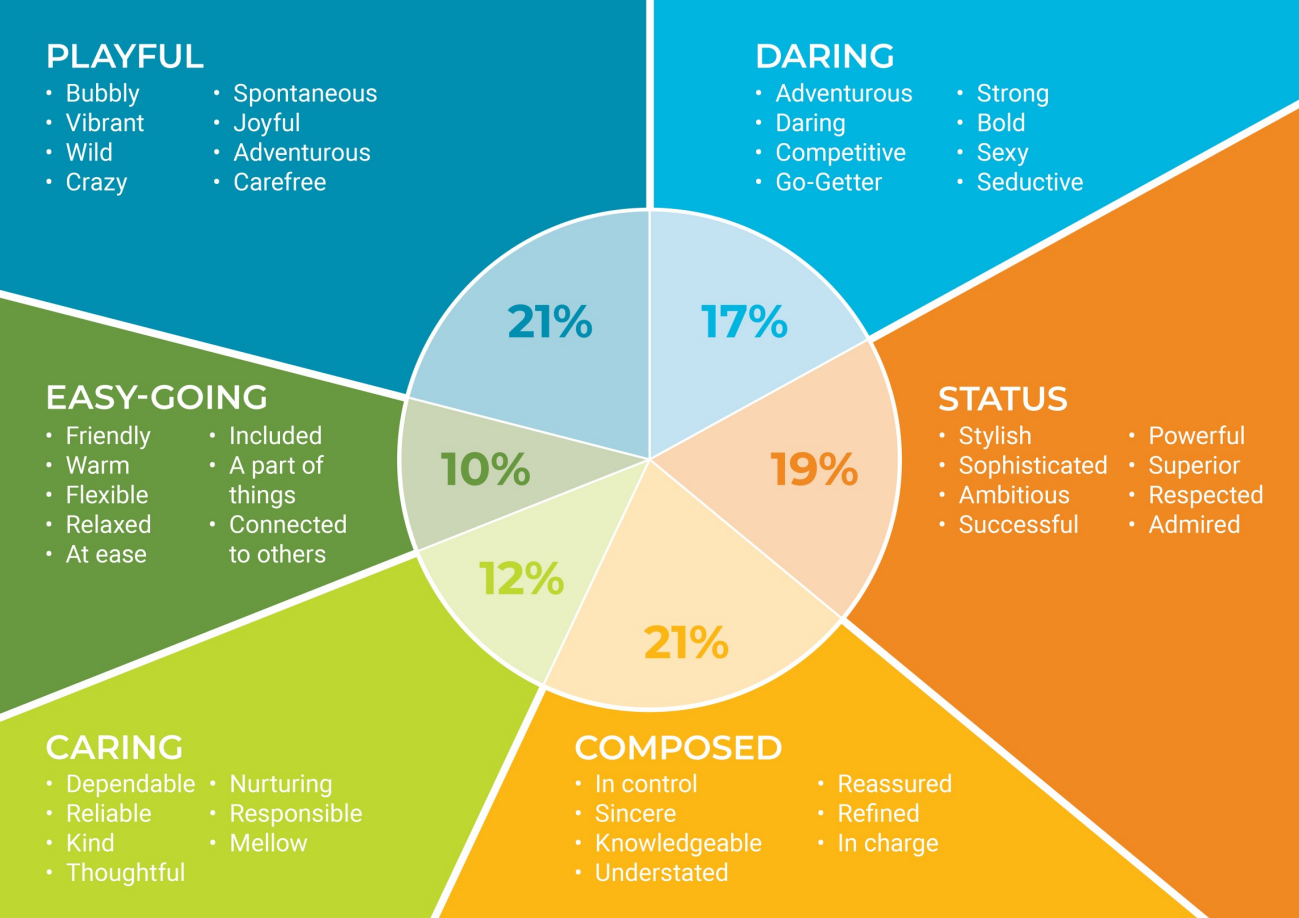
Results of audience segmentation research on Zimbabwe condom consumers.

PSI led a nationwide social marketing campaign to improve consumer targeting and promotion of PP. It repositioned and repackaged PP to increase demand among consumers with the ability to pay, improve emotional attachment to PP, and justify the price increase in the mind of the consumer. Repositioning efforts were based on audience segmentation research that revealed that PP should appeal to the “caring and easy going” audience segment to complement the positioning of public and commercial sector condoms, and avoid competing with the public and commercial sectors for the same group of consumers (Fig 3) [13]. This formative research was conducted prior to the study and was not evaluated as part of this intervention.

In addition, PSI modernized the packaging for PP, which included a tamper-proof seal and fun sex tips printed on the inside of packaging.

PSI used mass media to promote condoms and PP nationwide. There was a radio campaign with the slogan ‘Maximize the Moment.’ Over the years, PP had become synonymous with condoms and consumers would refer to PP when discussing the overall condom category. The ‘Maximize the Moment’ campaign gave the PP brand the potential to create a “halo effect” for the market and help promote the overall condom category. Mass media promotion also included social media posts on popular websites, like Facebook, Instagram, and Twitter. PSI leveraged specific events and holidays for media bursts to promote PP, popular DJs mentioned PP during their broadcasts, and PSI placed advertisements in the national press.

The following intensive promotional activities took place only in experimental districts.

- PSI increased PP product visibility in retail, stockist, and wholesale outlets, which included point-of-sale promotional materials like posters, bunting, category dividers, merchandising units, and shelf talkers. Merchandising units are temporary shelving units used to create a promotional product display. Shelf talkers are signs affixed to specific products on display, with the aim of drawing the attention of consumers to that product. Category dividers were also placed between different products in outlets to draw attention to different varieties of PP. Increasing product visibility was intended to prompt PP purchase and compete with other high-demand products, like alcohol, that retailers were likely to buy.
- Retailers received in-store promotions at stockist and wholesale outlets. To boost sales and strengthen PP brand loyalty, retailers who frequent stockists and wholesalers were encouraged to stock PP. This strategy was also intended to dissuade wholesalers from “delisting” PP, the practice of removing a product from registers due to low sales.
- Consumers received community-based condom promotional events, like PP ‘Pleasure Nights,’ in and around high-risk venues, where PP representatives would provide information and product demonstrations. These events provided “edutainment” to complement mass media and engage consumers on a personal level. Pleasure nights also created more business for traders, motivating them to increase condom orders. Non-stocking traders were also inspired to stock the product in anticipation of events.
- Traders in experimental districts received activities to improve their attitudes about PP given the new price increase and potential hits to their profit margins. Activities included 15 business review meetings to understand motivations and barriers to selling PP and 10 “sales blitzes” at key business centers to create excitement around the brand. Sales blitzes were also an opportunity to replenish stocks, improve product visibility in outlets, and prompt sales. In addition, PSI conducted 153 volume-based in-store promotions with stockists and wholesalers and two sales promotions to increase sales.

## Economic context

When the study was designed, the economic situation in Zimbabwe was stable. However, the economic context deteriorated rapidly in the lead up to the intervention. When the intervention was launched in March 2017, the economy had deteriorated to such an extent that several project activities were delayed or disrupted. This is important to consider when interpreting results on condom supply and demand.

Economic challenges included significant inflation, currency shortages, and shortages of consumer goods throughout 2017. Consumers and the trade had less purchasing power and faced hard economic choices, with the price of condoms equivalent to essential foodstuffs and other consumer goods due to inflation and shortages. The economic crisis also affected suppliers. Forex shortages made it nearly impossible for suppliers to import goods or raw materials, like paper and ink, critical for PP packaging and getting the product onto the market [14]. There were also periodic stock outs throughout the intervention.

## Methods

### Study population and sampling strategy

We randomized ten purposively selected districts and assigned them to two study groups. The five districts that did not receive the intensive promotional activities were: Chikomba, Gweru, Masvingo, Mutare, and Mwenezi (“the control group”). The five districts that received the intensive promotional activities were: Bulawayo, Chitungwiza, Harare, Makonde, Mt. Darwin (“the experimental group”). Districts were selected because they have a higher concentration of commercial distribution networks and higher HIV prevalence.

We collected data through pre-post consumer surveys and trader surveys in the ten districts. For the consumer survey, we used multi-stage cluster sampling to select respondents for baseline and follow-up surveys. At the first stage, we selected 100 enumeration areas from the control districts and 100 enumeration areas from experimental districts (90 urban/peri-urban and 10 rural in each). The second stage of sampling was a random selection of households. For each household, we interviewed an adult between 18 and 49 years. When there were multiple eligible respondents, we used a Kish grid to randomly select one respondent. To participate in the consumer survey, respondents must have had sex in the month preceding the survey and lived in the selected household for at least 6 months. We surveyed 3,400 consumers at baseline and at follow up.

For the trader survey, we conducted a census of all traders in the selected districts. The survey instrument collected information about traders’ past and current experience selling PP, current stock volumes, attitudes toward PP, and motivations for and barriers to stocking the brand. We conducted trader surveys at the same time as consumer surveys during baseline and follow up. We interviewed 604 traders at baseline and 628 traders at follow up. We conducted baseline consumer and trader surveys in November 2016, and the intervention began in March 2017. Follow-up surveys took place in November and December 2017.

### Data sources

In addition to the consumer surveys and trader surveys, we used routine sales and distribution data for PP and Panther condoms. For Panther distribution, we compared data from three different sources: National Condom Program at the Ministry of Health and Childcare, Chemonics (the prime on USAID’s Partnership for Supply Chain Management) and NatPharm (the national supply chain system).

### Sample size calculation

To calculate the sample size for each study group, we used PP brand preference (%) as the outcome of interest. Prior to baseline, brand PP preference was 59%. The desired minimum increase in brand preference was 5% with 80% power at 95% significance.

Since the study was conducted at the district level, we ensured that the sample size would account for intra-class correlation (ICC) within districts. No information on the magnitude of ICC was found in previous studies, so we used a design effect of 2 to appropriately account for clustering.

Based on these calculations, we identified a total minimum sample size of 3,392 individuals for the consumer survey at baseline. This yielded a total of 340 individuals interviewed per district in a total of 10 districts. The same sample size, 3,392, was used for the follow up survey.

For the trader survey, a census of all traders was conducted in the 10 districts and all eligible and consenting traders were included in the sample.

### Ethical approval

The Medical Research Council of Zimbabwe (MRCZ) ethics review board reviewed and approved the study. Its procedures adhere to the U.S. federal guidelines for human subjects as set forth in the Title 45, Part 46 of the Code of Federal Regulations. All study participants were consented before data collection.

### Statistical analysis

The primary outcomes for this study were: 1) PP sales volumes; and 2) Panther distribution volumes. We collected PP monthly sales data for each control and experimental district standardized by population size. We then tracked standardized sales data longitudinally to compare sales in control and experimental districts in the context of key intervention activities, like media bursts, extra promotions, and stock outs. As discussed above, Panther distribution data are tracked through three different sources, with differing methods for tracking geographic distribution. Since district-level data were unavailable, we standardized distribution data by population size. The estimates of distribution differed considerably between the different sources. Due to the differences between data sources, we created a top-line estimate of Panther distribution but could not confidently generate an estimate for the experimental and control districts. These analyses were conducted in Excel 2016.

Secondary outcomes included increased perceived availability of PP among consumers, increased coverage for PP, and self-reported increases in consumer and trade preferences for PP. Consumer outcomes were measured through a structured questionnaire administered to each eligible and consented individual who met the eligibility criteria. The questionnaire collected sociodemographic information, attitudes toward condoms, perceived availability of social marketed and public sector condoms, emotional attachment to the PP brand, intent to purchase PP, self-efficacy to negotiate condoms, and improved beliefs about condom efficacy. Multi-item scales were used to measure self-efficacy to negotiate condoms with partners, positive attitudes toward condoms (e.g. “The use of condoms can make sex more stimulating”), self-efficacy to negotiate condom use (e.g. “It is easy to suggest to a partner that he/she uses a condom”), beliefs about PP efficacy (e.g. “This brand of condoms is effective of HIV prevention”), and perceived availability of PP (e.g. “I know where to get this brand of condoms”). Scaled items were assessed on a four-point scale (strongly disagree, disagree, agree, strongly agree) and averaged to create a composite score for each category. The emotional attachment to PP measure was composed of several different sub-domains including brand loyalty to PP (e.g. “I will use Protector Plus the next time I use a condom”), brand quality (e.g. Protector Plus will not break during sex”), brand leadership (e.g. “Protector Plus is for people like me”), brand value (e.g. “Protector Plus is a good value for the price”), and brand personality (“Protector Plus is for couples who care about one another”) [15]. Intention to purchase PP was assessed using a binary outcome (yes or no). For trader outcomes, a structured questionnaire was administered to each eligible and consenting trader. The questionnaire collected information about traders’ past and current experience selling PP, current stock volumes, attitudes toward PP, and motivations for and barriers to stocking the brand. Multi-item scales were used to measure trader loyalty to the PP brand (e.g. “I will stock Protector Plus the next time I stock condoms”), perceptions of brand quality (e.g. “Protector Plus is a high-quality condom”), perception of brand leadership (“Protector Plus is for people like me”), and perception of brand value (e.g. “Protector Plus is a good value for the price”). Scaled items were assessed on a four-point scale (strongly disagree, disagree, agree, strongly agree) and averaged to create a composite score for each category. Trader satisfaction with sales volume and profits for PP varietals, whether they stocked PP, and whether they were willing to purchase from a wholesaler were assessed using a binary outcome (yes or no). The baseline and follow up of the trader survey occurred at the same time as the consumer surveys.

We used chi-square and t-tests for tabulations of consumer and trader characteristics in the baseline and follow up surveys to check the success of randomization and identify differences in group composition by socioeconomic or demographic factors. Next, we conducted univariate and multivariate difference-in-difference analyses between the baseline and follow up results for both the consumer and trader surveys. For the consumer survey, we included gender, marital status, and wealth quintile in the model to control for differences between groups and accounted for clustering by district. In the trader survey, we included outlet type in the model to account for differences between groups and clustering by district.

Outcome=α(ExposedGroup)+γ(AfterIntervention)+δ(ExposedGroup*AfterIntervention)

We used Stata v.13.1 for data analysis. Results are presented as significant at the 5% level.

## Results

### Respondent characteristics

#### Consumer characteristics

Table 1 shows characteristics of consumers by study group and time. There were 3,403 respondents at baseline and 3,395 respondents at follow up.

**Table 1:** Consumer characteristics at baseline and follow up surveys.

Variable	Baseline			Follow-Up		
	Control (n = 1,677)	Experimental (n = 1,726)	p-value	Control (n = 1,680)	Experimental (n = 1,715)	p- value
Gender						
Male	677 (40%)	694 (40%)	0.924	550 (33%)	754 (44%)	<0.001
Female	1,000 (60%)	1,032 (60%)		1,130 (67%)	961 (56%)	
Age						
18–24	532 (32%)	494 (29%)	0.143	465 (28%)	435 (25%)	0.263
25–29	425 (25%)	442 (26%)		423 (25%)	409 (24%)	
30–34	293 (18%)	307 (18%)		319 (19%)	350 (20%)	
35–39	238 (14%)	250 (15%)		252 (15%)	265 (16%)	
40–44	108 (6%)	149 (9%)		144 (9%)	154 (9%)	
45–49	81 (5%)	84 (5%)		77 (5%)	102 (6%)	
Wealth Quintile						
Poorest	281 (17%)	434 (25%)	<0.001	269 (16%)	376 (22%)	<0.001
Poor	252 (15%)	360 (21%)		385 (23%)	363 (21%)	
Medium	317 (19%)	333 (19%)		390 (23%)	341 (20%)	
Wealthy	406 (24%)	278 (16%)		382 (23%)	280 (16%)	
Wealthiest	421 (25%)	321 (19%)		254 (15%)	355 (21%)	
Residence						
Rural	149 (9%)	190 (11%)	0.039	155 (9%)	186 (11%)	0.117
Urban	1,528 (91%)	1,536 (89%)		1,525 (91%)	1,529 (89%)	
Marital Status						
Married/cohabitating	978 (58%)	1,101 (64%)	<0.001	1,032 (61%)	1,093 (64%)	0.592
Never married	538 (32%)	426 (25%)		456 (27%)	447 (26%)	
Widowed	40 (2%)	46 (3%)		40 (2%)	32 (2%)	
Divorced	60 (4%)	65 (4%)		64 (4%)	57 (3%)	
Separated	61 (4%)	88 (5%)		88 (5%)	86 (5%)	

At baseline, women made up 60% of the control group respondents and 60% of experimental group respondents. At follow up, women represented 67% of the control group and 56% of the experimental group. In addition, there were differences in the proportion of married/cohabitating respondents between groups: 58% of the control group were married or cohabitating at baseline compared to 64% in the experimental group. Since gender and marital status can impact condom preferences and purchasing behavior, we controlled for gender and marital status during analysis.

There were also significant differences between the groups in terms of wealth quintile. At baseline, 32% of respondents in the control group were in the bottom two wealth quintiles as compared to 46% of respondents in the experimental group. We therefore controlled for wealth quintile in the final analysis, as socioeconomic status impacts ability to pay for condoms.

#### Trader characteristics

Table 2 shows trader characteristics by study group. In the control group, informal stores selling alcohol, called “bottle stores” (22% and 23%) and stores selling a variety of everyday products, called “general dealers” (25% and 26%) were the most prevalent types of outlets at baseline and follow up. In the experimental group, supermarkets (28%) and bottle stores (21%) were the most prevalent at baseline. At follow up, pharmacies (24%) were the most prevalent type of outlet in the experimental group. Pharmacies represented 6% of the control group at baseline and 12% at follow up, versus 7% of the sample at baseline and 24% at follow up in the experimental group. Given the differences in group composition by owner type, we controlled for outlet type during analyses.

**Table 2:** Trader characteristics in baseline and follow up surveys.

Variable	Baseline			Follow Up		
	Control (n = 209)	Experimental (n = 395)	p- value	Control (n = 245)	Experimental (n = 383)	p- value
Outlet type						
Wholesaler	10 (5%)	23 (6%)	0.027	9 (4%)	11 (3%)	<0.001
Stockist	0 (0%)	7 (2%)		2 (1%)	0 (0%)	
Supermarket	42 (20%)	110 (28%)		23 (9%)	76 (20%)	
Bottle store	45 (22%)	81 (21%)		57 (23%)	77 (20%)	
Night club	9 (4%)	17 (4%)		11 (4%)	21 (5%)	
Service station	7 (3%)	3 (1%)		7 (3%)	25 (7%)	
General dealer	53 (25%)	63 (16%)		64 (26%)	30 (8%)	
Pharmacy	12 (6%)	29 (7%)		29 (12%)	92 (24%)	
Beerhall	14 (7%)	26 (7%)		11 (5%)	9 (2%)	
Sports bar	14 (7%)	32 (8%)		14 (6%)	30 (8%)	
Other	3 (1%)	4 (1%)		18 (7%)	12 (3%)	
Location						
Rural	38 (18%)	7 (2%)	<0.001	34 (14%)	15 (4%)	<0.001
Urban	171 (82%)	388 (98%)		211 (86%)	368 (96%)	

### Primary outcomes: PP sales volumes and Panther distribution volumes

Our two hypotheses were that the rebranding and repositioning efforts for PP would lead to an increase in consumer demand for PP and willingness of traders to stock PP. The primary outcome was monthly sales volumes of PP. We hypothesized that both control and experimental districts would see sales of PP rebound to the same levels prior to the price increase. We also hypothesized that sales in experimental districts would exceed those of control districts as a result of intensive promotional activities in the experimental districts.

**Fig 4:**
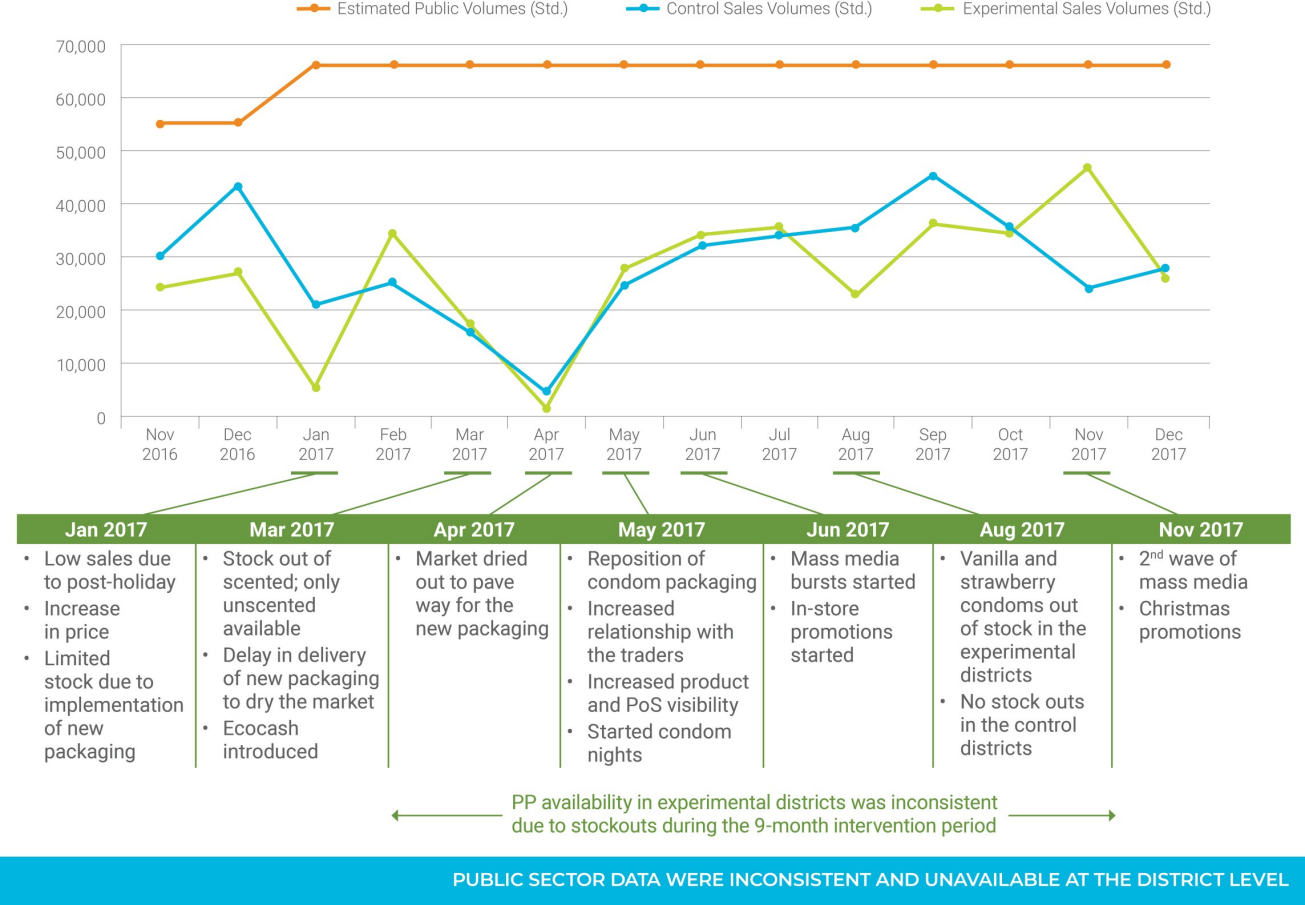
PP sales volumes and Panther distribution by month (standardized volumes by 100,000 population).

Results are presented in Fig 4. For PP, we standardized volumes by population to compare estimates of monthly sales in experimental and control areas across all study months, since the overall population of the experimental districts was much larger than the control districts. Overall, PP sales rebounded after the price increase and repositioning, and remained strong through 2017. The standardized sales volumes of PP mirrored each other quite closely in the experimental and control groups of the study, suggesting that the intervention had no impact on PP sales.

Decreases in March and April 2017 are explained by PSI’s efforts to eliminate the old PP packaging in the pipeline and allow rebranded PP condoms to move quickly to the shelf once introduced. By May 2017, the new PP packaging had been fully introduced and intervention activities, such as strengthening relationships with traders, increasing PP visibility at points of sale, and PP Pleasure Nights with consumers, had begun. Sales of PP rebounded in May 2017 and continued into June and July, corresponding with the beginning of mass media promotions for PP. The decrease in sales in the experimental groups in August 2017 is most likely due to a stock out of several PP Scented variants.

Periodic stock outs during the nine-month study period were largely due to forex limitations, which hampered local printing suppliers from importing paper at the required volumes and resulted in printing delays for the new packaging and smaller-than-expected quantities were produced. There were no stock outs of PP Scented in the control groups because the sales estimates reflected the stock required to serve demand on the ground. Based on the trend in sales, we believe that volumes would have continued to increase in both study groups had there not been stock outs due to paper and ink shortages for packaging. We also believe that, had we monitored the intervention longer than 9 months, we would have seen increases in sales that were adversely affected by economic “shocks” during the study period.

For Panther condoms, no monthly distribution figures were available, so we standardized the annual figure from the MOH to reflect the population of the study areas and divided it between the 12 study months to obtain the results presented in Fig 4. Volumes are similar to Panther distribution figures for 2016 and averaged 66,354 per month. Inconsistent data and the inability to track Panther condom distribution at a district level made it impossible to measure the impact of the intervention in experimental and control districts.

### Secondary outcomes

#### Consumer survey results

To further test our first hypothesis—that consumer attachment to PP would increase as a result of intensive promotional activities—we conducted a consumer-based survey. We used this survey to measure the impact of the intervention on consumer preferences and attitudes (Table 3).

**Table 3:** Differences in self-reported consumer outcomes for PP condoms.

Variable	Control		Experimental		Diff-in-diff	p
	Baseline	Follow Up	Baseline	Follow Up		
Emotional attachment to the PP Original brand	3.01	2.95	2.95	3.04	0.147	**0.007**
Emotional attachment to the PP Scented brand	3.01	2.96	2.95	3.01	0.117	**0.025**
Intention to purchase PP condoms in the next month	0.34	0.30	0.36	0.31	-0.002	0.996
Self-efficacy to negotiate condoms with partners	3.06	2.95	3.01	2.99	0.084	0.389
Positive attitudes toward condoms	2.67	2.73	2.61	2.75	0.073	0.174
Improved beliefs about PP Original condom efficacy	3.00	2.96	2.95	3.08	0.173	**0.001**
Improved beliefs about PP Scented condom efficacy	2.98	2.98	2.93	3.07	0.142	**0.004**
Increased perceived availability of PP Original among consumers	2.84	2.74	2.83	2.77	0.036	0.584
Increased perceived availability of PP Scented among consumers	2.80	2.77	2.75	2.74	0.015	0.813

Intensive promotional activities were successful in creating greater emotional attachment to PP and improving beliefs about condom efficacy in the experimental group as compared to the control group. We observed an increase in self-reported emotional attachment to both PP Original and PP Scented condoms among the experimental group as compared to the control group (p = 0.007 for PP Original and p = 0.025 for PP Scented). There was also a significant increase in beliefs about the efficacy of both PP Original and PP Scented condoms in the experimental group as compared to the control group (p = 0.001 for PP Original and p = 0.004 for PP Scented).

There were, however, no noticeable differences between the experimental and control groups for the other secondary outcomes, including intention to purchase condoms, self-efficacy to negotiate condoms with partners, positive attitudes towards condoms, and perceived availability of PP condoms.

#### Trader survey results

To test the second hypothesis—that intensified trade relations would lead to traders being more willing to stock PP—we conducted a survey among traders. Results are presented in Table 4. The intervention had no significant impact on outcomes in the experimental group when compared to the control group. There was no overall change in the proportion of traders with PP in stock between the study groups during the follow-up survey. There were also no significant differences between baseline and follow up on perceptions of the PP brand or satisfaction with sales and profits.

**Table 4:** Differences in self-reported trader outcomes for PP condoms.

Variable	Control		Experimental		Diff-in-diff	p
	Baseline	Follow Up	Baseline	Follow Up		
PP currently stocked	0.91	0.98	0.97	0.98	-0.059	0.386
Perception of brand quality among traders to PP	3.29	3.10	3.22	3.11	0.086	0.524
Feelings of brand loyalty among traders to PP	3.28	3.10	3.20	3.10	0.085	0.482
Perception of brand leadership among trader to PP	3.28	3.18	3.28	3.19	0.015	0.909
Perception of brand value of PP among traders	3.09	2.99	3.10	2.94	-0.055	0.592
Satisfied with profit from PP Original	0.75	0.69	0.80	0.82	0.077	0.338
Satisfied with profit from PP Scented	0.78	0.88	0.72	0.91	0.100	0.223
Satisfied with PP Original sales volume	0.81	0.73	0.73	0.78	0.130	0.296
Satisfied with PP Scented sales volume	0.82	0.83	0.71	0.89	0.168	0.125
Willing to purchase PP from a wholesaler	0.52	0.60	0.50	0.64	0.064	0.410
Purchases PP from stockist/wholesaler	0.47	0.44	0.44	0.53	0.123	0.102

**Fig 5:**
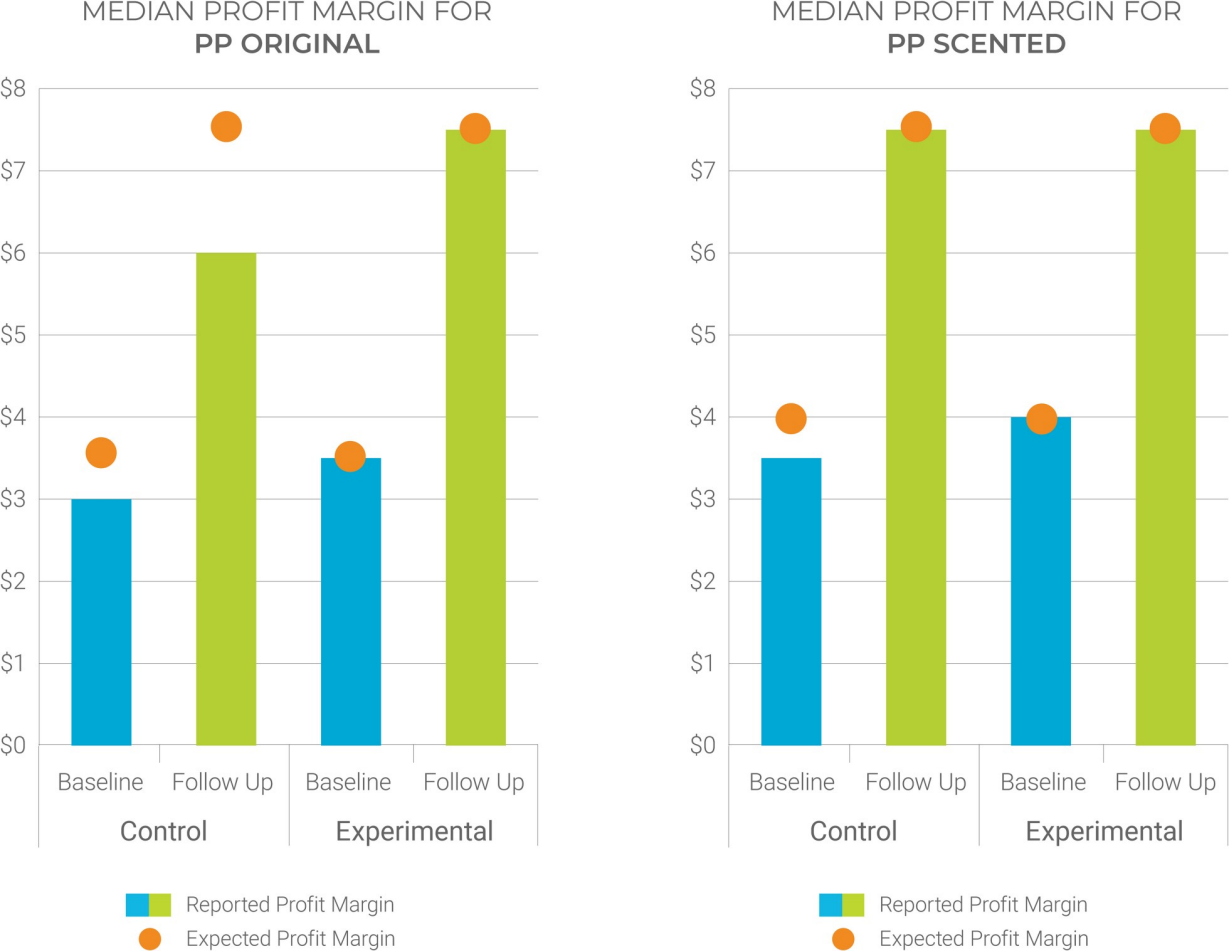
Alignment between actual and expected profit margins for PP condoms.

To explore the profit-driven nature of retailers and gains in efficiency in the new PP pricing structure, we examined the alignment between actual and expected profit margins (Fig 5). Prior to the price increase, some retailers had been selling PP at more than the recommended consumer price and earning as much as 140% profit margins. After standardizing all PP variants at $0.50, we see an alignment between median profit margins as reported by retailers and their expected profit margins. These results combined with those in Table 4 indicate that interventions for creating improved relationships with traders were unnecessary: ensuring acceptable profit margins is what matters most to the trade.

## Discussion

In this study, we tested the impact of an intensive CSM intervention and support to the public sector on overall market supply and increased demand for condoms to highlight where strategic donor investments can impact condom programming and condom markets. The study showed that the intervention was effective at increasing consumer emotional attachment to PP condoms and consumer beliefs about condom efficacy.

Study findings demonstrate where international donor and government investments can impact condom programming and condom markets. There were also several lessons learned for improving condom coverage and collaboration between the public, social marketing, and commercial sectors as well as areas for future research. Strategic investments for strengthening condom markets include:

- **Formative research to understand consumer preferences and where each market sector is best positioned to meet demand**. Prior to the study, PSI conducted market research and worked with the public and commercial sectors to segment audiences for Zimbabwe’s public, social marketed, and commercial condoms. This was critical for targeting condoms to the appropriate audiences and offers promise for creating a healthier and stronger market. During the study, consumers assigned higher value to PP after the price increase and targeted marketing. Appropriate segmentation minimizes crowding out and maximizes market efficiency by ensuring free and subsidized condoms benefit consumers with no or less ability to pay. Investments in condom markets should ensure that the different market sectors do not compete for the same consumers. Market research should be used to position condom brands in a complementary—rather than competitive—fashion.
- **WTP studies to identify the appropriate price point for condoms.** PP supported a substantial price increase: a 150% price increase for PP Original and a 67% price increase for PP Scented despite a challenging economic context. This suggests that social marketed condoms can maintain volumes and absorb price increases when the right investments are made in branding and marketing, and when the price is based on WTP intelligence. When examining WTP data, decision-makers must consider the tradeoffs a price increase presents for consumers and the trade. Setting condom prices according to consumer WTP stabilizes the market and builds trader confidence, which eliminates the need to over invest in trade relations to maintain volumes.
- **Intensive demand generation activities to increase demand for condoms and ultimately increase use.** The intensive promotional activities, which included enhanced product visibility and community-based promotions, led to more emotional attachment to the PP brand and better perceptions of condom efficacy. Increasing demand requires genuine insight and a consumer-centred approach where the promise of the product is clear and market actors use multiple methods to generate market-wide demand for condoms. Mass media alone cannot trigger purchase for complex products like condoms: there is need to engage the consumer on a personal level.
- **Effective market facilitation that looks beyond the social marketing sector to work in partnership with public and commercial sector actors**. The principles of good social marketing can be applied to the public sector, but it takes time to gain buy-in, facilitate change, and shift focus from distribution figures to more meaningful analytics like condom use, equity, and source of supply. Work with the public sector can also be slow: it requires support on market stewardship and time for buy-in. Stewardship also extends to the commercial sector and will require motivating commercial actors to participate in markets. During the study, a new commercial condom at the $0.50 price point was introduced in Zimbabwe. The main challenge for the commercial sector will be economies of scale, since the commercial sector cannot lower prices and cover costs without higher volumes.
- **Investments in improved market analytics.** Market intelligence is essential for informing decision-making and identifying where market-level changes are needed to increase impact. Inconsistent public sector data made it impossible to assess the impact of activities for improving public sector condom distribution. In the case of Zimbabwe, market actors need a more accurate picture of the condom volumes pulled from central stores down to a district and site level. Market actors should also shift their focus from condom distribution figures to more meaningful analytics like condom use, equity, and source of supply.

This study and the overall intervention revealed areas for future research to inform international donor and government investments in condom programming and market strengthening.

- **More frequent and inexpensive measures for condom use**. Condom use is typically measured by use at last sex rather than consistent condom use, which is a stronger indicator of how often and how many condoms are being used. Standard surveys like Demographic and Health Surveys (DHS), Bio-behavioral Surveys for Key Populations, and The Population-based HIV Impact Assessment survey are common data sources for condom use, but they can be expensive and infrequent. Investments in more frequent measures of condom use are needed. Alternatively, modeling could be used. Other health areas, like family planning, use modeling to estimate modern contraceptive use on an annual basis [16]. For HIV prevention, a similar modeling process could be used, but it would need to be standardized and supported by the larger prevention community. Measurement among key populations, like female sex workers and clients, men-who-have-sex-with-men, and people who inject drugs would also need to be included to determine the effectiveness of market investments on condom use.
- **Better measures of equity.** Experimental study designs could be used to answer questions about equity and to determine if international donor and government subsidies are targeting the right consumers across wealth, geography, and gender categories. The DHS and other behavioral surveys contain questions about household assets, but the number of questions could be reduced for ease of data collection, implementation, and analysis. Ideally, equity would also be calculated by sector source of supply.
- **Behavioral factors associated with condom use**. In addition to investing in formative research, international donors and governments should support repeated cross-sectional surveys and from representative samples of target audiences to identify factors associated with condom use. Factors like awareness, self-efficacy, and risk perception are based on grounded theory and analyzed through logistic regression. Results provide information on how to best design programs to increase condom use.

### Limitations

Limitations of this study include a compressed intervention period of nine months, which was too short to measure the desired behavior change for condom purchasing and use, especially given the rapidly deteriorating economic environment. The price increase and PP rebranding were nationwide and, although we could randomize districts and isolate the intensive promotional activities to experimental areas, we did not measure awareness of the general PP campaign. As a result, we could not determine if general campaign awareness was similar across groups. Likewise, we did not measure intensity or frequency of exposure to the nationwide or experimental interventions nor particular elements of the PP repositioning, like just the packaging change or just the new slogan. Testing consumer and trader responses by different exposure levels or different campaign elements would be appropriate for future research.

The rapid rate of inflation and currency shortages likely affected the willingness and ability of consumers and traders to buy or stock PP condoms. Moreover, forex shortages impacted the ability of PP manufacturers and marketers to obtain needed supplies, which led to persistent condom shortages during the intervention and study period.

Since PP was already the preferred brand on the Zimbabwe market, a rebound in sales or positive perceptions of the PP rebranding could have reflected pre-existing consumer preferences. Unfortunately, we were unable to fully operationalize the work with the public sector, and inconsistent and poor quality data for Panther condom distribution made it impossible to measure the impact of the intervention on the public sector. Future TMA interventions and studies that include the public sector will need to include longer timelines and flexible strategies to see changes at this level.

## Conclusions

This study demonstrates how targeted donor investments can improve a CSM program and strengthen the overall condom market. The intervention increased program efficiency while increasing demand and strengthening supply. Given more time and a better economic context, improvements in intention to buy and condom use could also improve. When a disciplined CSM approach is used, the market benefits: subsidies can be better targeted, branded products can appeal to the right audiences, and room can be made for the commercial sector to enter the market.
